# Supplementary material for: Interplay of Quantum Size Effect and Tensile Strain on Surface Morphology of β‑Sn(100) Islands
Source: ACS Nano. 2026 Mar 2;20(10):8289–97. doi: 10.1021/acsnano.5c14019 (PMC13001075; doi:10.1021/acsnano.5c14019)
Supplement: Supplementary file 1 [file nn5c14019_si_001.pdf]

Supporting Information

Interplay of Quantum Size Effect and Tensile Strain on Surface Morphology of β-Sn(100) Islands

Bing Xia[1,2,6], Xiaoyin Li[3,6], Hongyuan Chen[1], Bo Yang[1], Jie Cai[1], Stephen Paolini[2], Zihao Wang[2], Zi-Jie Yan[2], Hao Yang[1,4,5], Xiaoxue Liu[1,4,5], Liang Liu[1,4,5], Dandan Guan[1,4,5], Shiyong Wang[1,4,5], Yaoyi Li[1,4,5], Canhua Liu[1,4,5], Hao Zheng[1,4,5], Cui-Zu Chang[2], Feng Liu[3], and Jinfeng Jia[1,4,5]

[1]TD Lee Institute and School of Physics and Astronomy, Shanghai Jiao Tong University, Shanghai 200240, China

[2]Department of Physics, The Pennsylvania State University, University Park, PA 16802, USA

[3]Department of Materials Science and Engineering, University of Utah, Salt Lake City, Utah 84112, USA

[4]Hefei National Laboratory, Hefei 230088, China

[5]Shanghai Research Center for Quantum Sciences, Shanghai 201315, China

[6]These authors contributed equally: Bing Xia and Xiaoyin Li

Corresponding authors: cxc955@psu.edu (C.-Z. C); fliu@eng.utah.edu (F. L.); jfjia@sjtu.edu.cn (J. J.).

Contents:

I. Supplementary Figures

II. Supplementary Tables

III. Supplementary Text

1. **Phase transition during Sn growth on graphene**
2. **Extraction of PCPS values**
3. **More discussion of the effects of the graphene substrate and the underlying α-Sn layers on the surface energy of β-Sn(100) islands**
4. **More discussion on flat surface energies of β-Sn(100) films under different biaxial tensile strains**
5. **More discussion on the mechanisms of surface pattern formation and strain relaxation**
6. **More discussion on surface morphology**

References

I. Supplementary Figures

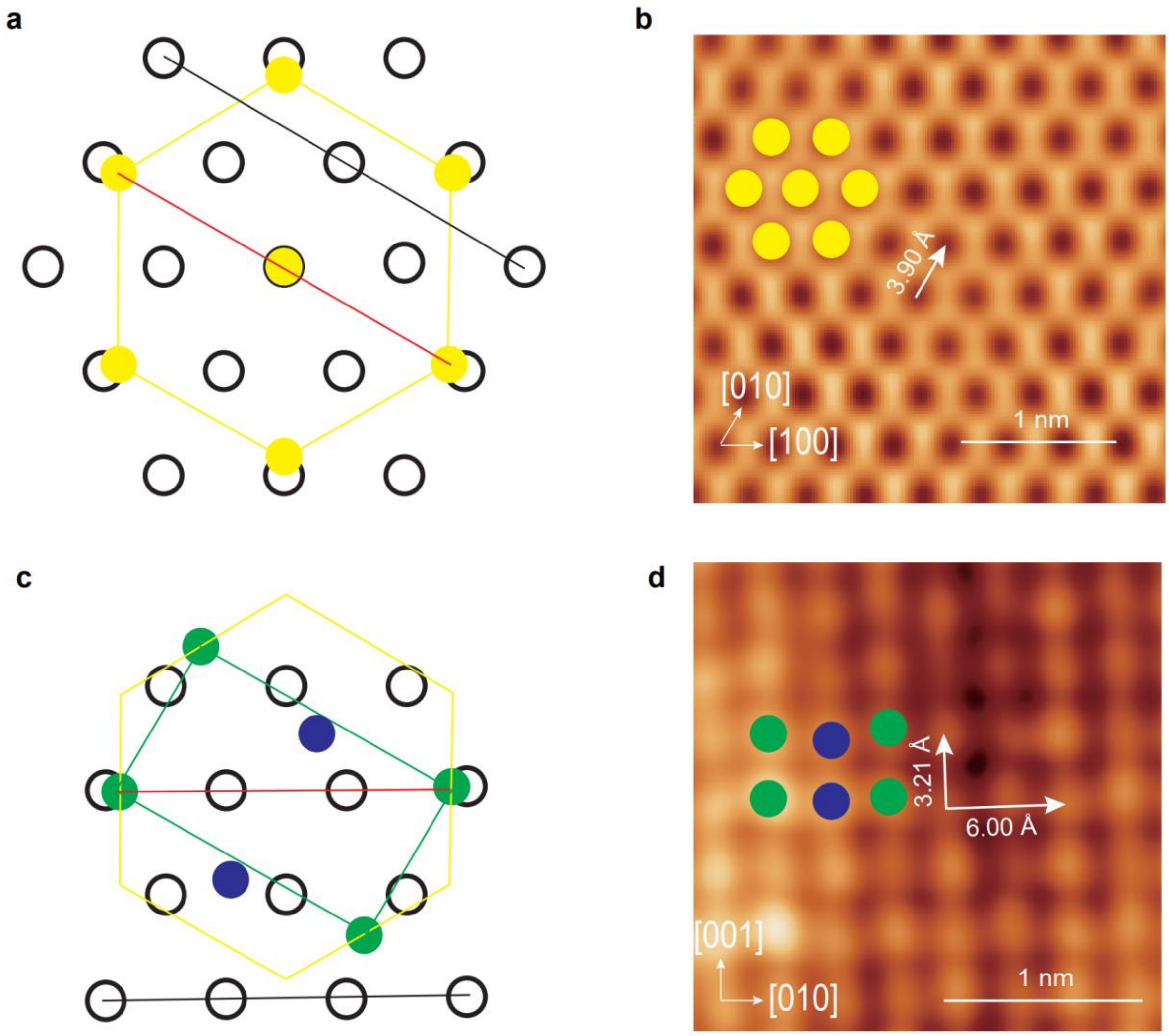

Figure S1| Epitaxial growth mode analysis. a, c, Schematic illustrations of the epitaxial orientation of α-Sn (**a**) or β-Sn (**c**) on the graphene substrate. The black hollow circles denote the center of honeycomb structures, yellow solid circles denote α-Sn atoms with yellow solid lines representing lattice spacing in the hexagonal lattice. Green and blue solid circles denote β-Sn atoms with green solid lines representing lattice spacing in the rectangular lattice. Red solid lines indicate in-plane Sn atomic distances, while black solid lines denote in-plane graphene atomic distances. These two distances are well matched to each other, thereby facilitating epitaxial growth. **b, d,** Atomic resolution STM images of a α-Sn island with a height of 680 pm ($N = 2$) and a β-Sn island with a height of 3.6 nm ($N = 12$) respectively. The yellow, green, and blue solid circles represent Sn atoms with the same designations as in (**a**) and (**c**).

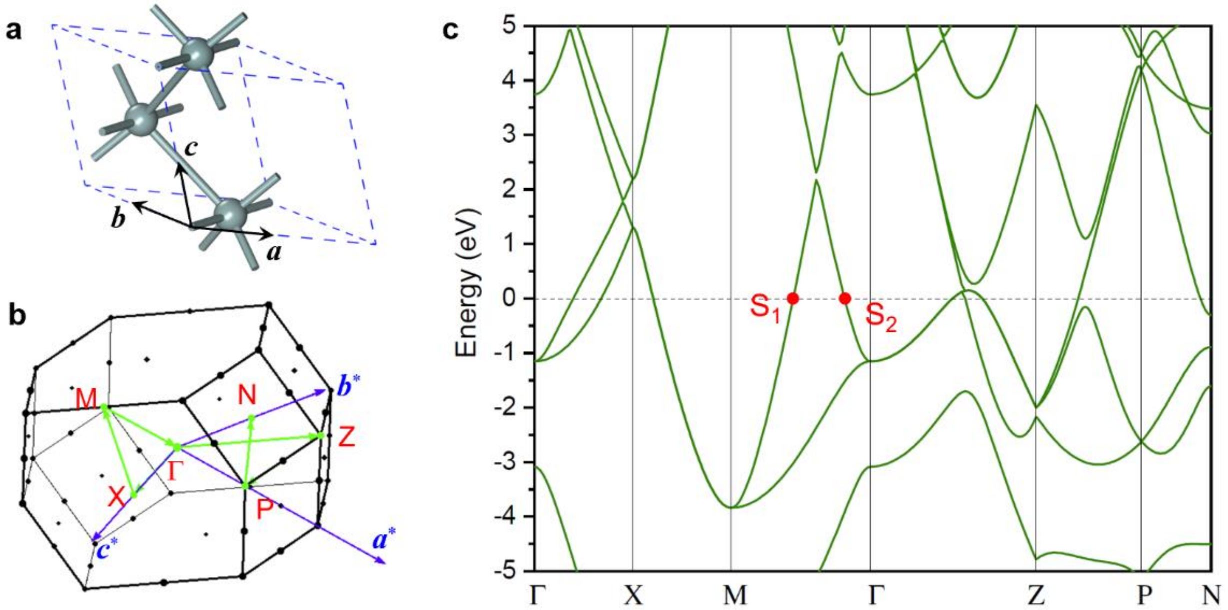

Figure S2| Electronic band structure of bulk b-Sn. a, Real-space primitive cell. **b,** Reciprocal space Brillouin zone of bulk β-Sn. **c,** Electronic band structure of bulk β-Sn along high-symmetric *k*-paths. The corresponding high-symmetric k-points are labeled in (**b**). The M-G path corresponds to the growth direction of β-Sn(100) islands, where one obtains a Fermi wavelength of k_{F1} = 0.4714 $Å^{-1}$ measured from M point and a Fermi wavelength of k_{F2} = 0.1925 $Å^{-1}$ measured from G point (the crossing points between electronic bands and the Fermi energy are marked as S_1 and S_2 respectively). Given the interlayer spacing d = 2.97 Å, the oscillation periodicity for QSE is estimated to be $\Delta N_1 = p/(k_{F1}d) = 2.24$ or $\Delta N_2 = p/(k_{F2}d) = 5.49$.

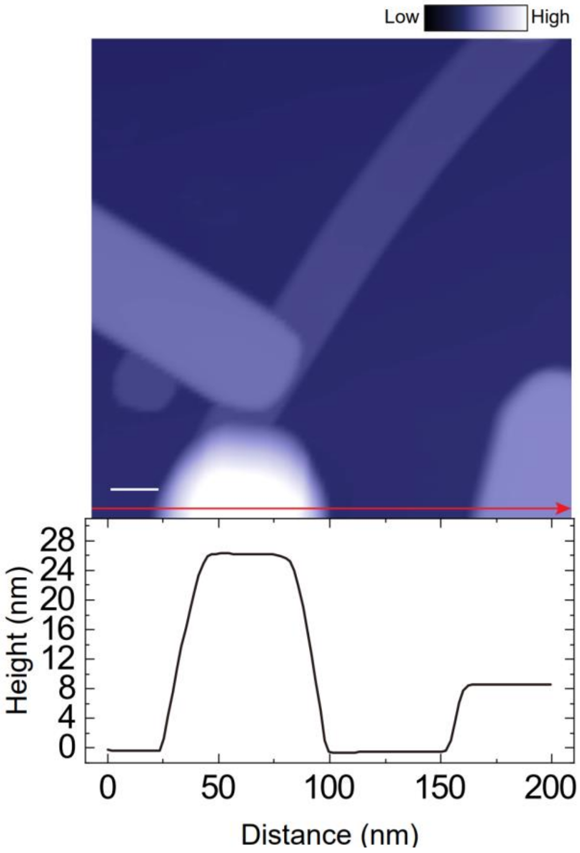

Figure S3| Surface morphology of a β-Sn(100) island with *N* = 90. Large scale STM images (200 × 200 nm^2) of β-Sn(100) islands on graphene-terminated 6H-SiC(0001) (V_B = 1.5 V and I_t = 0.02 nA). The bottom panel shows a height profile along the red arrow in the top STM image. Scale bar: 20 nm. All STM measurements are performed at $T \sim 4.2$ K.

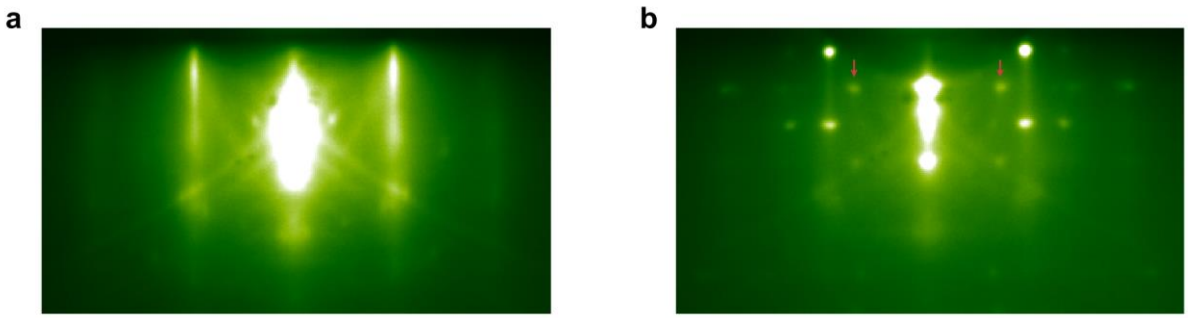

Figure S4| RHEED patterns. a, Graphene-terminated 6H-SiC(0001). **b,** β-Sn films with a nominal $N = 20$. The spacing between the diffraction spots marked by red arrows corresponds to the space between (001) crystal planes, indicating a lattice constant of ~320 pm along the [001] direction. The incident electron beam is aligned along the [010] direction.

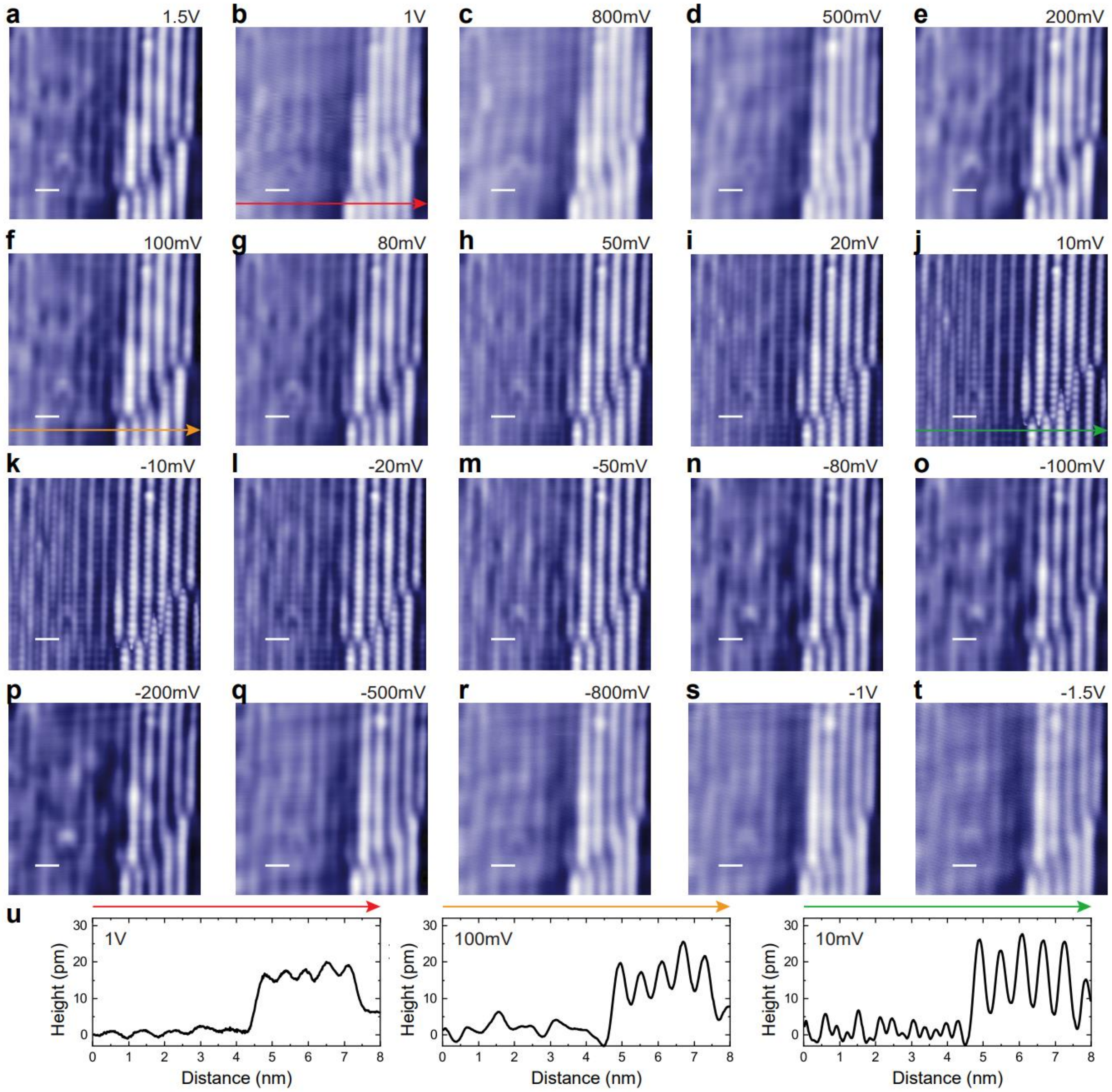

Figure S5| STM images of a β-Sn(100) island with N = 16 acquired under different sample bias V_B. a-t, STM images of a transition region between flat and patterned surfaces, with V_B varying from 1.5 V (**a**) to -1.5 V (**t**). The V_B value for each image is indicated in the top right corner. **u,** Height profiles along the arrows in the STM images in (**b**, **f**, **j**), respectively. Scale bar: 1 nm (**a**-**t**). All STM measurements are performed at $T \sim 4.2$ K.

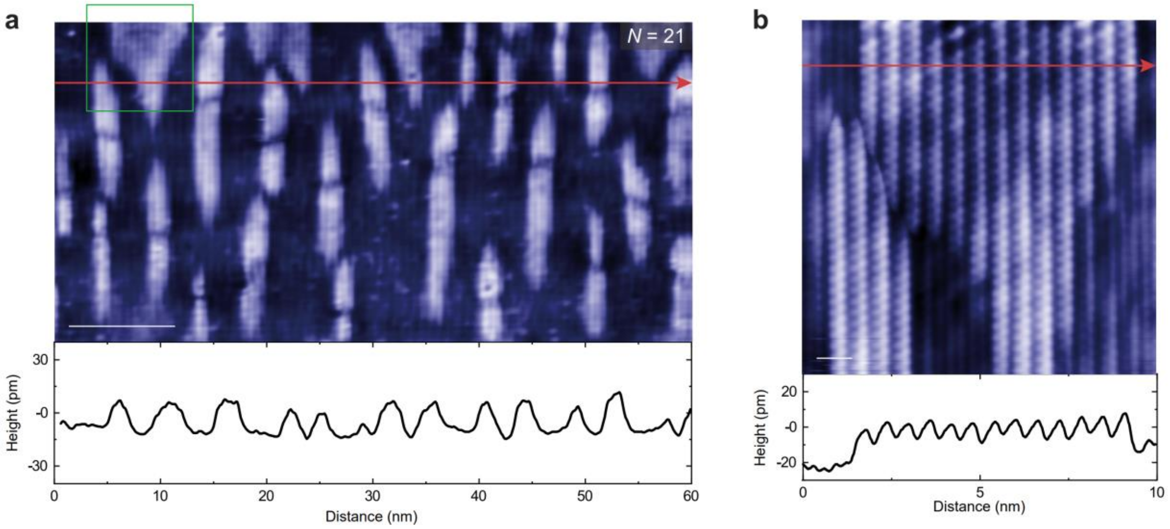

Figure S6| Estimation of the PCPS value for a β-Sn(100) island with *N* = 21. a, Large scale STM image (V_B = 1 V and I_t = 0.1 nA). **b,** Atomic resolution STM image of the green square region in (**a**) (V_B =10 mV and I_t =8 nA). The bottom panels in (**a**) and (**b**) show height profiles along the red arrows in the corresponding STM images. Scale bars: 10 nm (**a**); 1 nm (**b**). All STM measurements are performed at $T \sim 4.2$ K.

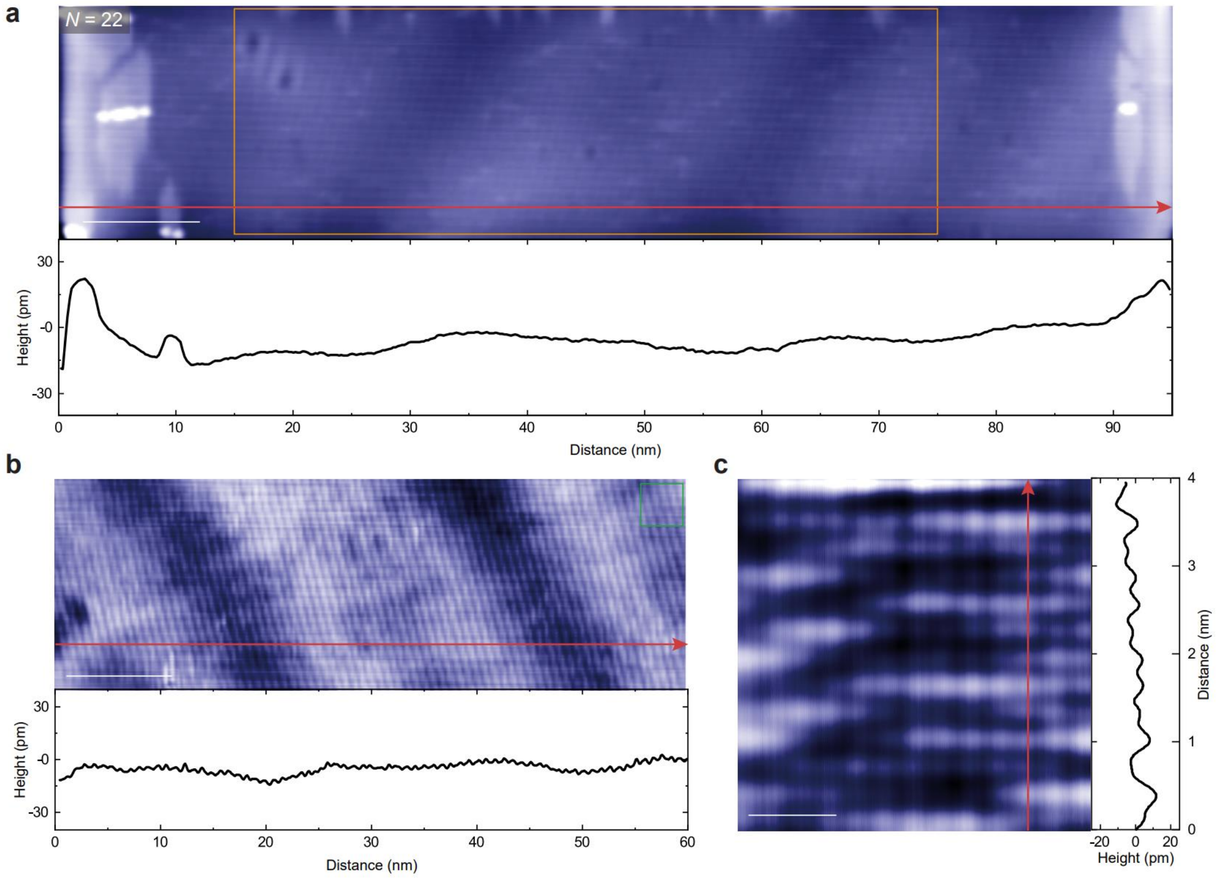

Figure S7| Estimation of the PCPS value for a β-Sn(100) island with N = 22. a, Large scale STM image (V_B = 1 V and I_t = 0.1 nA). **b,** Zoomed-in STM image of the orange rectangular region in (**a**) (V_B =100 mV and I_t =1 nA). **c,** Atomic resolution STM image of the green square region in (**b**) (V_B = 10 mV and I_t = 8 nA). The STM image in (**c**) is rotated by 90° relative to (**b**). The bottom panels in (**a**,**b**) and the right panel in (**c**) show height profiles along the red arrows in the corresponding STM images. Scale bars: 10 nm (**a**,**b**); 1 nm (**c**). All STM measurements are performed at T ~ 4.2 K.

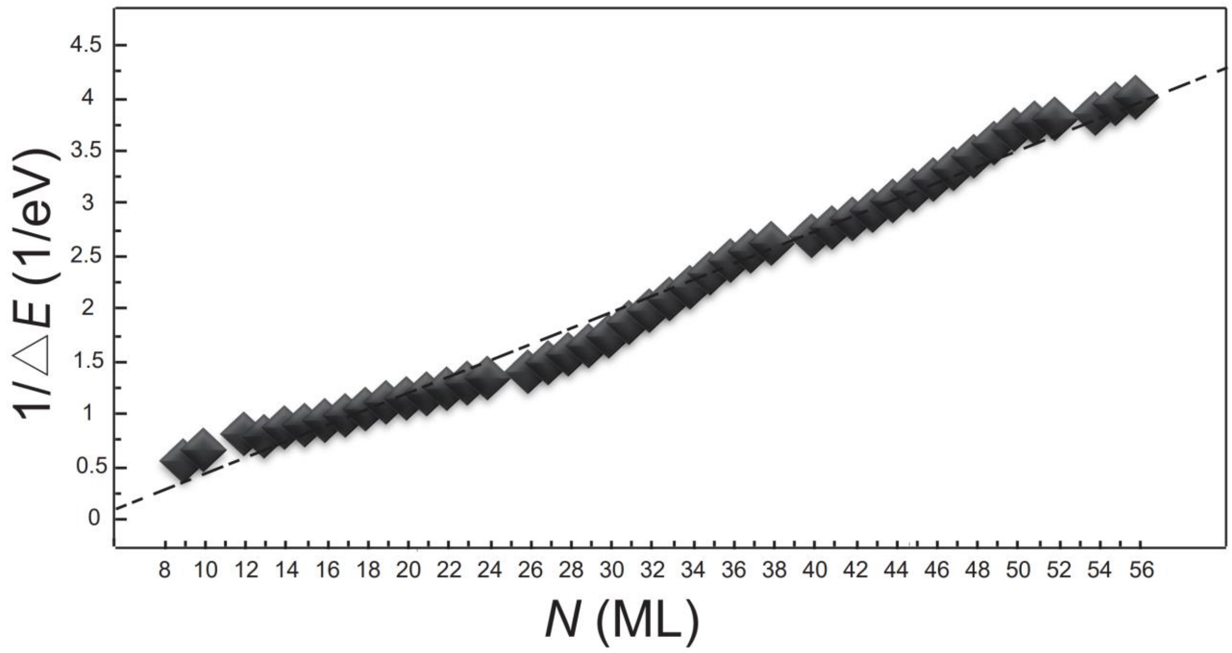

Figure S8| Inverse energy spacing between LUQWS and HOQWS in β-Sn(100) island with different *N*.

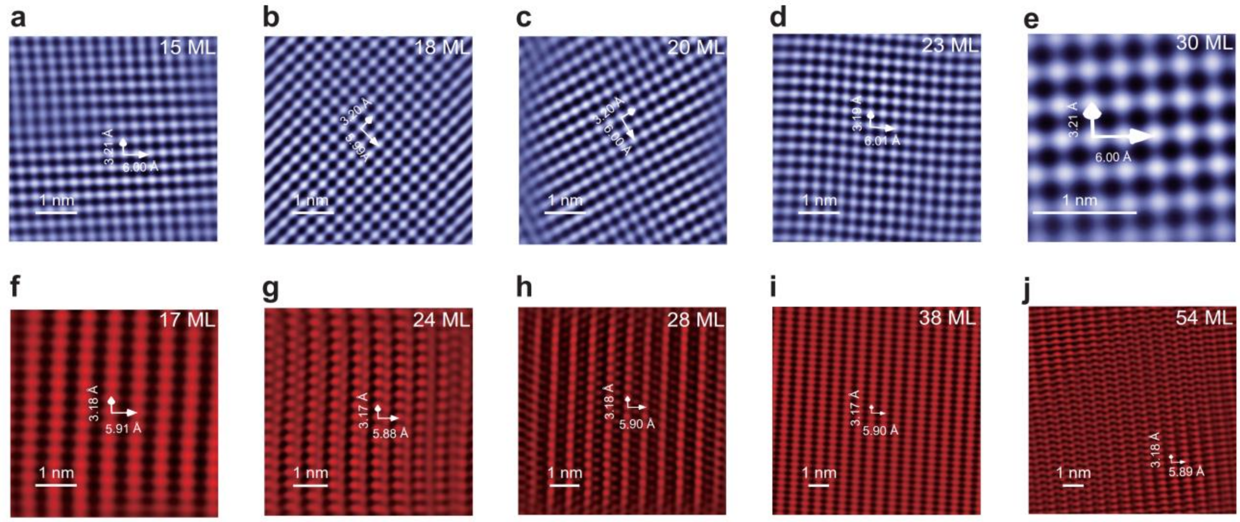

Figure S9| Atomic resolution STM images of the β-Sn(100) islands with different *N*. a-e, Atomic resolution STM images of the flat surfaces of islands with $N = 15$, $N = 18$, $N = 20$, $N = 23$, and $N = 30$, respectively. **f-j,** Atomic resolution STM images of the patterned surfaces of islands with layer numbers $N = 17$, $N = 24$, $N = 28$, $N = 38$, and $N = 54$, respectively. The STM topographic images were acquired in constant current mode and processed using WSXM software. All STM measurements are performed at $T \sim 4.2$ K.

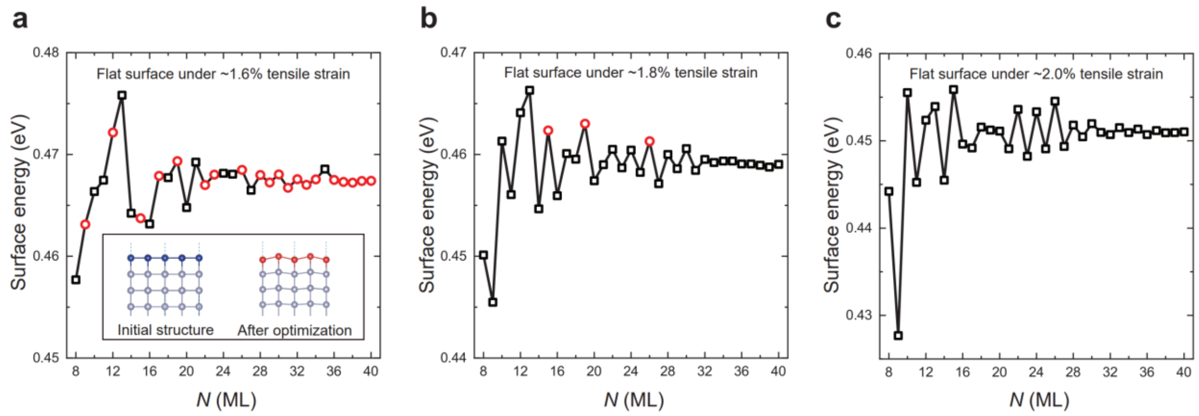

Figure S10| Surface energies of flat β-Sn(100) films under different biaxial tensile strains. a, β-Sn(100) films under ~1.6% biaxial tensile strain. Black squares denote surfaces that remain flat before and after structural relaxation. Red circles denote surfaces initially flat but relaxed into patterned configurations. For this strain level, thick films with initially flat surfaces relax into patterned configurations, indicating that ~1.6% tensile strain is insufficient to stabilize flat surfaces. **b**, β-Sn(100) films under ~1.8% biaxial tensile strain. Only three *N* (highlighted by red circles) show relaxation from initially flat to patterned surfaces. **c**, β-Sn(100) films under ~2% biaxial tensile strain. All β-Sn(100) films retain flat surfaces after structural relaxation.

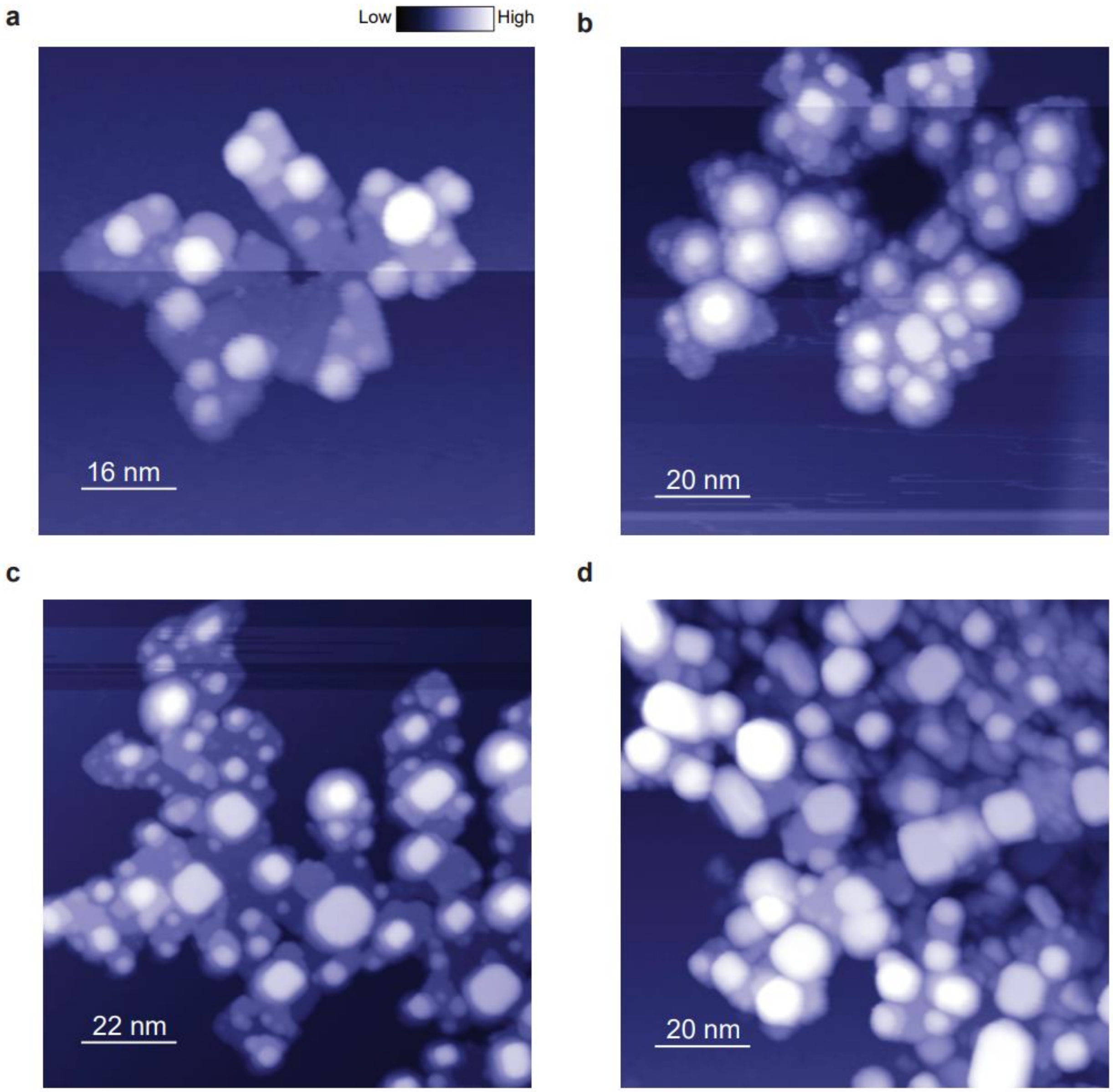

Figure S11| Topography of Sn after different growth-measurement rounds. The number of growth-measurement rounds is one (**a**), two (**b**), four (**c**), and five (**d**) respectively. Sn was grown at room temperature ($T = 300$ K) with a low growth rate for 10 minutes in every round of growth. All STM measurements are performed at $T \sim 4.2$ K.

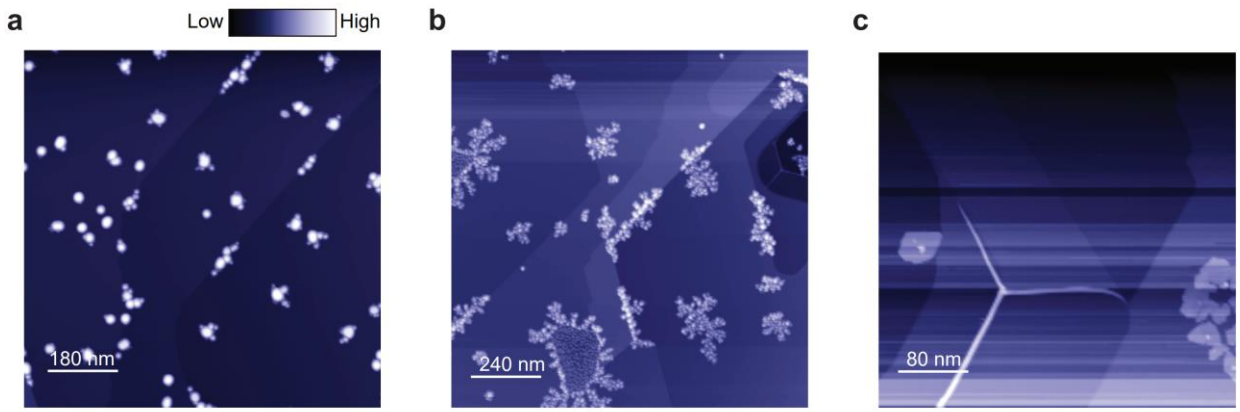

Figure S12| Topography of Sn under different substrate temperatures. The substrate temperatures are T = 20 K (**a**), T = 300 K (**b**), and T = 373 K (**c**). All measurements are derived from a single growth process conducted at a low growth rate. All STM measurements are performed at T ~ 4.2 K.

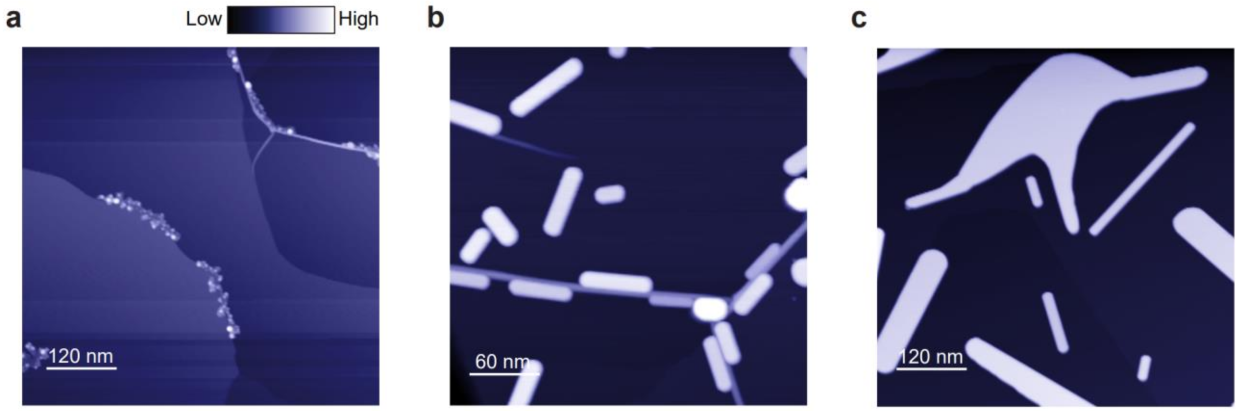

Figure S13| Topography of Sn under different growth rates and growth time. The growth conditions include low rate and short time (**a**), high rate and long time (**b**), and higher rate and longer time (**c**). All measurements are derived from a single growth process at room temperature ($T = 300$ K). All STM measurements are performed at $T \sim 4.2$ K.

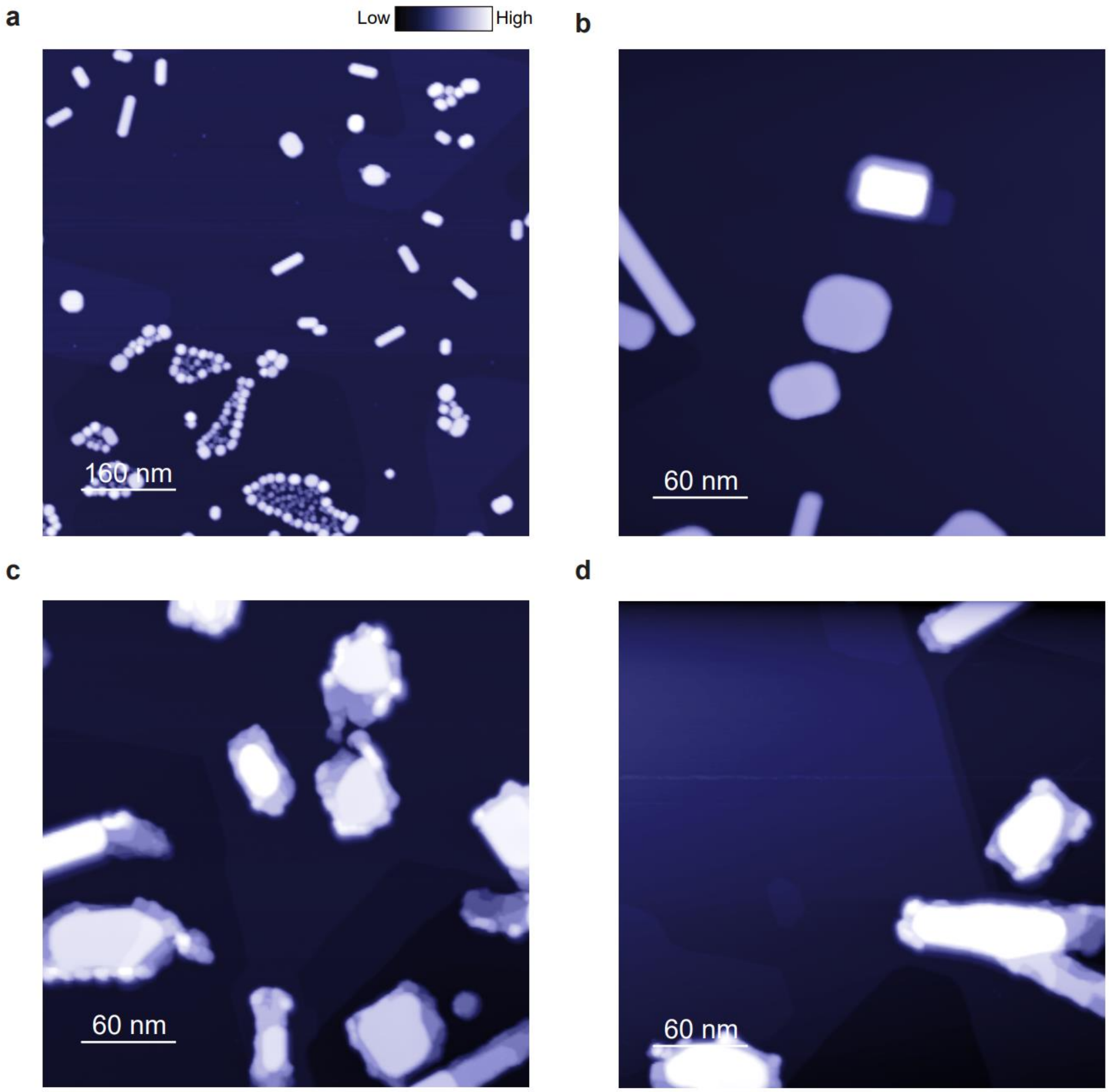

Figure S14| Topography of β-Sn(100) islands before and after annealing. a, b, Topography of as-grown β-Sn(100) islands. **c, d,** Topography of β-Sn(100) islands after annealing at room temperature for two weeks. All STM measurements are performed at $T \sim 4.2$ K.

II. Supplementary Tables

Table S1| Summary of data for all analyzed islands. The table includes layer number, thickness, sample count, averaged PCPS, sample size, PCPS for each sample, and standard deviation of PCPS. In the table, "None" indicates no data was collected, while "Single" denotes that the standard deviation is not applicable due to a single data set.

layer number	thickness (nm)	number of sample#	averaged PCPS	size(length nm * width nm)			PCPS of each sample			standard deviation of PCPS
				#1	#2	#3	#1	#2	#3	
9	2.673	2	0	54*16	30*20	none	0	0	none	0
10	2.97	2	0	45*45	45*30	none	0	0	none	0
11	3.267	0	none	none	none	none	none	none	none	none
12	3.564	2	0	30*30	20*20	none	0	0	none	0
13	3.861	3	8.83	47*27	30*30	30*20	8.4	11.2	6.9	2.18
14	4.158	1	0	45*17	none	none	0	none	none	single
15	4.455	3	26.77	40*26	42*20	50*24	26.9	27.1	26.3	0.42
16	4.752	1	19	45*17	none	none	19	none	none	0
17	5.049	3	28.1	30*20	38*12	31*13	35	24.3	25	5.99
18	5.346	3	4.43	55*8	40*10	30*10	9.8	3.5	0	4.97
19	5.643	3	43.23	50*7.32	20*20	50*10	47.5	45.8	36.4	5.98
20	5.94	3	3.1	50*7.32	30*10	30*5	9.3	0	0	5.37
20	6.237	3	20.87	60*30	50*6	41*6	29.5	19.7	13.4	8.11
22	6.534	3	15.2	95*20	48*7	24*6	10.2	12.5	22.9	6.77
23	6.831	3	60.07	55*10	41*16	60*15	58.2	68.3	53.7	7.48
24	7.128	3	42.9	60*15	65*13	60*15	44	42.4	42.3	0.95
25	7.425	0	none	none	none	none	none	none	none	none
26	7.722	2	61.15	18*8	15*6	none	55.6	66.7	none	7.85
27	8.019	2	50	18*8	38*7	none	55.6	44.4	none	7.92
28	8.316	2	64.05	64*27	36*10	none	73.7	54.4	none	13.65
29	8.613	2	80.05	40*20	42*20	none	75	85.1	none	7.14
30	8.91	2	81.75	25*37	28*10	none	93.5	70	none	16.62
31	9.207	2	71.7	38*25	100*18	none	73.4	70	none	2.4
32	9.504	1	73.8	40*40	none	none	73.8	none	none	single
33	9.801	1	100	20*20	none	none	100	none	none	single
34	10.098	1	100	8*10	none	none	100	none	none	single
35	10.395	1	94.3	50*30	none	none	94.3	none	none	single
36	10.692	1	92	30*30	none	none	92	none	none	single
37	10.989	1	98.4	50*50	none	none	98.4	none	none	single
38	11.286	1	95	60*20	none	none	95	none	none	single
39	11.583	0	none	none	none	none	none	none	none	none
40	11.88	1	93.8	55*20	none	none	93.8	none	none	single
41	12.177	1	100	70*40	none	none	100	none	none	single
42	12.474	1	97.5	40*18	none	none	97.5	none	none	single
43	12.771	1	98.3	60*40	none	none	98.3	none	none	single
44	13.068	1	100	20*20	none	none	100	none	none	single
45	13.365	1	94.1	25*25	none	none	94.1	none	none	single
46	13.662	1	100	20*20	none	none	100	none	none	single
47	13.959	1	95.8	20*20	none	none	95.8	none	none	single
48	14.256	1	100	50*20	none	none	100	none	none	single
49	14.553	1	96	45*25	none	none	96	none	none	single
50	14.85	1	100	10*10	none	none	100	none	none	single
51	15.147	1	100	40*40	none	none	100	none	none	single
52	15.444	1	100	15*15	none	none	100	none	none	single
53	15.741	0	none	none	none	none	none	none	none	none
54	16.038	1	100	20*20	none	none	100	none	none	single
55	16.335	1	100	10*10	none	none	100	none	none	single
56	16.632	1	100	20*20	none	none	100	none	none	single
61	18.117	1	100	20*10	none	none	100	none	none	single
64	19.008	1	100	25*25	none	none	100	none	none	single
80	23.76	1	100	10*10	none	none	100	none	none	single
90	26.73	1	100	15*15	none	none	100	none	none	single

Table S2| Summary of the formation mechanism of abnormal surface patterns.

Thickness N	Strain ε	Origin of strain	Percentage surface coverage of flat surface	Surface energy of flat surface E_{flat}	E_{flat} VS $E_{patterned}$	Percentage surface coverage of patterned surface	Surface energy of patterned surface $E_{patterned}$
$9 \le N < 11$	> +2%	Expansion to match substrate	~ 100%	$E_{flat}(\varepsilon=+2\%)$ lowest	<<	~ 0%	$E_{patterned}(\varepsilon=0\%)$ highest
$12 \le N < 25$ even	~ +1.7%	Slightly away from substrate	~ 90%	$E_{flat}(\varepsilon=+2\%)$ oscillating to low value	≤	~ 10%	$E_{patterned}(\varepsilon=0\%)$ oscillating near middle value
$12 \le N < 25$ odd	~ +1.7%	Slightly away from substrate	~70%	$E_{flat}(\varepsilon=+2\%)$ oscillating to high value	≥	~ 30%	$E_{patterned}(\varepsilon=0\%)$ oscillating near middle value
$26 \le N < 33$	~ +1.7% → ~ 0%	Further away from substrate	~ 60% → ~ 20%	$E_{flat}(\varepsilon=+1\%)$ converging to high value with micro oscillation	>	~ 40% → ~ 80%	$E_{patterned}(\varepsilon=0\%)$ converging to low value with micro oscillation
$N \ge 33$	~ 0%	Far enough from substrate to form bulk-like structure	~ 0%	$E_{flat}(\varepsilon=0\%)$ converging to high value	>>	~ 100%	$E_{patterned}(\varepsilon=0\%)$ converging to low value

III. Supplementary Text

1. Phase transition during Sn growth on graphene

For a thin Sn island with $N = 2$ on graphene-terminated 6H-SiC(0001), an α-Sn layer with a height of ~680 pm and a hexagonal lattice structure are observed (Figure S1b). In contrast, for thicker Sn islands with $N = 12$ on the same substrate, a β-Sn layer with a height of ~3.6 nm and a tetragonal lattice structure are observed (Figure S1d). A similar α-to-β-Sn phase transition of Sn islands on Si(111) has been reported in a prior study [8]. For $N \leq 3$, the Sn island adopts the α-Sn phase, primarily due to the same lattice symmetry between α-Sn and Si(111). A α-to-β-Sn phase transition occurs near $N = 3.5$, driven by the lower bulk free energy of β-Sn relative to that of epitaxially stabilized α-Sn. For $N \geq 4$, the upper layers fully convert to β-Sn , while the bottom 3 ML α-Sn remain unchanged. Since graphene-terminated 6H-SiC (0001) and Si(111) share the same hexagonal surface symmetry, Sn islands grown on graphene-terminated 6H-SiC (0001) are expected to follow the same mechanism as Sn islands on Si(111).

A quantitative analysis of the in-plane lattice constant reflects the strain of each phase. The atomic resolution image of the α phase reveals a compressed lattice constant $\boldsymbol{a}_{\alpha\text{-Sn}(111)} = 3.90 \pm 0.02$ Å, compared to the theoretical 4.60 Å, indicating in-plane contraction to match graphene's atomic spacing (Figure S1a,b). While that of β phase shows an expanded lattice constant $\boldsymbol{b}_{\beta\text{-Sn}(100)} = 6.00 \pm 0.02$ Å, compared to the theoretical 5.90 Å, reflecting tension as β-Sn adjusts to α-Sn's lattice (Figure S1c,d). The origin of tension here is similar to that observed in other epitaxial systems where the substrate lattice is larger than the film lattice [9-14]. This tensile strain, characteristic of epitaxial Sn growth on graphene, gradually relaxes with increased thickness, forming high-layer β-Sn islands closer to equilibrium. Additionally, during cooling, tensile strain accumulates in Sn due to its higher thermal expansion coefficient (~2.3 10^{-6}/K) relative to the SiC

substrate (~0.4 10^{-6}/K), similar to Ge/Si(100) systems [15-17].

2. Extraction of PCPS values

In this work, we define patterned surfaces as regions exhibiting a height difference exceeding 10 pm relative to flat surfaces in STM images acquired at V_B < 1 V (Figure 2c to 2h). Since the absolute height difference depends sensitively on the tip resolution, we perform atomic resolution STM measurements across a transition region between flat and patterned surfaces of a β-Sn(100) island with N = 16 under different V_B (Figure S5). For -1.5 V ≤ V_B ≤ 1.5 V, although the absolute height contrast decreases with reduced tip resolution (Figure S5a to 5t), a height difference exceeding 10 pm between flat or patterned surfaces is clearly resolved in all height profiles (Figure S5u). These tests confirm that a height difference threshold of more than 10 pm provides a reliable criterion for distinguishing the flat or patterned surfaces in our STM images of β-Sn(100) islands with different N.

To clarify how the PCPS value is extracted for each N, we take two representative β-Sn(100) islands with N = 21 and N = 22 as examples (Figures S6 and S7). For the N = 21 island (Figure S6), the patterned surface area is ~531.7 nm^2 out of a total island area of ~1800 nm^2, corresponding to a PCPS value of ~29.5%. In contrast, for the N = 22 island (Figure S7), the surface is predominantly flat, with a patterned area of ~193.6 nm^2 within a total island area of ~1900 nm^2, yielding a PCPS value of ~10.2%. To ensure generality and accuracy, we collect data from multiple islands for each N, with at least three in Range II, two in Ranges I and III, and one in Range IV (Table S1). For each N, the PCPS value is extracted by calculating the patterned surface fraction for each island and then averaging over islands with the same N. The resulting N-dependent PCPS curve is plotted with the standard deviation as the error bar (Figure 3a).

3. More discussion of the effects of the graphene substrate and the underlying α-Sn layers

on the surface energy of β-Sn(100) islands

In our DFT calculations, we do not include the bottom α-Sn layers and just consider only free-standing β-Sn islands with varying *N*. By comparing our DFT results with experimental observations, we can infer the roles of the graphene substrate and the bottom thin α-Sn layers. The substrate roles on the surface energy of Pb films have been systematically investigated in a prior study [18]. Across all studied cases, including free-standing Pb films and Pb films grown on semiconducting or metallic substrates, a robust bilayer oscillation of the surface energy is consistently observed. While the presence of the substrate can modify the beating pattern and the decay thickness of the oscillation, it does not eliminate the fundamental odd-even oscillatory behavior. For Sn islands grown on graphene-terminated 6H-SiC (0001), directly simulating the full Sn island structure is technically challenging, particularly due to the formation of the bottom α-Sn layers and the gradual strain relaxation with increasing *N*. Nevertheless, guided by the prior studies on Pb films, we expect that the graphene substrate and the bottom α-Sn layers similarly affect only the beating pattern and the decay thickness of the oscillation, without altering the essential odd-even oscillation behavior of the surface energy.

4. More discussion on flat surface energies of β-Sn(100) films under different biaxial tensile strains

For β-Sn under biaxial tensile strain, we fix in-plane lattice constants $\boldsymbol{b}$ and $\boldsymbol{c}$, vary out-of-plane constant $\boldsymbol{a}$ along [100], and relax atomic positions to find the optimal $\boldsymbol{a}_{\mathbf{strain}}$ that minimizes the total energy. We find that, in some cases, the surfaces cannot retain its initially imposed morphology after structural relaxation (Figure S10).

To examine the influence of strain resolution, we also calculate flat surface energies for β-Sn(100) islands with different *N* under biaxial tensile strains of ~ 1.6% and ~ 2.0% (Figure S10).

We find that the results of the ~ 1.8% and ~ 2.0% strain cases are very similar, with three N values under 1.8% strain relaxing into patterned surfaces. This tendency is more pronounced in the ~ 1.6% strain result (Figure S10a), where more than half of the studied N cannot maintain the initially imposed flat surface after structural relaxation. These results indicate that, under ~ 1.6% biaxial tensile strain, the surface energies of patterned and flat surfaces become comparable.

By combining these results with the two limiting cases in our strain setting, specifically, the flat surface under ~ 2.0% biaxial tensile strain (Figure S10c) and at the equilibrium case (blue curve in Figure 5a), we construct a coarse evolution of the flat surface energy as a function of biaxial tensile strain. This evolution is roughly consistent with the gradual release of tensile strain with increasing N in β-Sn(100) islands. Therefore, we infer that the overall strain states of the β-Sn(100) islands in Range I, II, and IV correspond to ~ 2.0%, ~ 1.8%, and the equilibrium case, respectively.

5. More discussion on the mechanisms of surface pattern formation and strain relaxation

Range I ($N = 9$ and $N = 10$): β-Sn islands experience over 2% tensile strain due to epitaxial growth on graphene, leading to ISRE. The lower surface energy of flat surfaces under a large tensile strain stabilizes an entirely flat island topography.

Range II ($12 \leq N \leq 24$): Slight strain release (~1.7%) occurs as the top surface separates from the substrate. Flat surface energy oscillates with thickness: even layers favor flat surfaces (flat surface is relatively stable). In contrast, odd layers favor patterned surfaces (flat surfaces are relatively unstable), resulting in mixed topography with near-equilibrium patterned surfaces.

Range III ($26 \leq N \leq 33$): Most tensile strain is released (~1%), and patterned surfaces become more stable due to lower surface energy. Incomplete energy convergence with micro-oscillations sustains a mixed surface, preventing complete flatness.

Range IV ($N \geq 33$): Strain is fully released, allowing bulk-like structures. Patterned surfaces dominate, as they are energetically favored in the near-equilibrium state.

We emphasize that strain relaxation occurs through a change in the relative population of the two surface patterns rather than by gradually relaxing the in-plane lattice constants. Specifically, the flat and patterned surfaces represent two surface stress domains with distinct intrinsic surface stress tensors, which may vary with film thickness. Energy minimization of surface stress domains can be achieved by optimizing domain populations [19-21], which depends on the relative surface energies, stress tensors, domain boundary energy, and strain of the domains. Ideally, given these parameters, one could calculate the domain population for fixed two-dimensional domain shapes [20]; however, this is too complex in our case, as all these parameters vary with thickness, and the domain shapes are not fixed.

6. More discussion on surface morphology

6.1 The number of growth-measurement rounds

In every growth-measurement round, Sn is grown at a low speed for 10 mins, and then we measure the sample at T ~4.2 K. In the low temperature measurement processes, the Sn surface may relax itself as discussed in strain analysis in Supplementary Text 1, which indicates the next round of growth may happen on low-temperature-relaxed Sn surface rather than on graphene surface. Figure S11c,d shows stacked Sn islands from multiple rounds, where upper-layer islands may deviate from the bulk structure due to the following rounds of epitaxial growth on low-temperature-relaxed Sn, rather than on pure graphene. To ensure consistent surface morphology, the experimental data in the text are collected from single-growth samples at a higher growth rate.

6.2 Substrate temperature

Figure S12 illustrates the impact of substrate temperature on Sn island formation. At low

temperatures, sparse β-phase island clusters form due to reduced mobility (Figure S12a), while room temperature promotes dense β-phase clusters with higher mobility (Figure S12b). Patterned regions appear in both cases. At high temperatures, Sn forms sparse α-phase islands or rod-like structures at graphene steps, driven by α-phase stability or ultra-high Sn mobility (Figure S12c). All data are collected from room-temperature ($T = 300$ K) samples to ensure PCPS comparability.

6.3 Growth rate and growth time

Figure S13 highlights how growth conditions influence Sn morphology. At low growth rates and short durations, Sn forms particles instead of islands (Figure S13a). Increasing the growth rate and time leads to independent Sn islands (Figure S13b), while further increases produce larger or contiguous islands (Figure S13c). Despite these variations, the β-Sn(100) island topography in Figure S13b,c remains consistent, indicating that growth rate and time minimally impact surface morphology. All data are collected from samples grown under high growth rates and long-duration conditions, as in Figure S13b,c, to ensure PCPS comparability.

6.4 Annealing time

After two weeks of annealing at room temperature, small clusters form around larger islands, and the island surfaces become smoother, as shown in Figure S14c,d, indicating complete strain release through external relaxation. All experimental data in the text are collected from as-grown islands without any annealing to focus on the internal relaxation process of the β-Sn(100) system (Figure S14a,b).

References

1. Kresse, G.; Furthmuller, J. Efficient iterative schemes for ab initio total-energy calculations using a plane-wave basis set. *Phys. Rev. B* **1996,** 54, 11169-11186.
2. Blochl, P. E. Projector augmented-wave method. *Phys. Rev. B* **1994,** 50, 17953-17979.
3. Perdew, J. P.; Burke, K.; Ernzerhof, M. Generalized gradient approximation made simple. *Phys. Rev. Lett.* **1996,** 77, 3865-3868.
4. Perdew, J. P.; Burke, K.; Ernzerhof, M. Generalized gradient approximation made simple (vol 77, pg 3865, 1996). *Phys. Rev. Lett.* **1997,** 78, 1396-1396.
5. Monkhorst, H. J.; Pack, J. D. Special Points for Brillouin-Zone Integrations. *Phys. Rev. B* **1976,** 13, 5188-5192.
6. Fiorentini, V.; Methfessel, M. Extracting convergent surface energies from slab calculations. *J. Condens. Matter Phys.* **1996,** 8, 6525-6529.
7. Lu, G. H.; Huang, M. H.; Cuma, M.; Liu, F. Relative stability of Si surfaces: A first-principles study. *Surf. Sci.* **2005,** 588, 61-70.
8. Roldan Cuenya, B.; Doi, M.; Keune, W. Epitaxial growth and interfacial structure of Sn on Si(111)-(7x7). *Surf. Sci.* **2002,** 506, 33-46.
9. Dejneka, A.; Tyunina, M.; Narkilahti, J.; Levoska, J.; Chvostova, D.; Jastrabik, L.; Trepakov, V. A. Tensile strain induced changes in the optical spectra of $SrTiO_3$ epitaxial thin films. *Phys. Solid State* **2010,** 52, 2082-2089.
10. Nichols, J.; Terzic, J.; Bittle, E. G.; Korneta, O. B.; De Long, L. E.; Brill, J. W.; Cao, G.; Seo, S. S. A. Tuning electronic structure via epitaxial strain in Sr_2IrO_4 thin films. *Appl. Phys. Lett.* **2013,** 102, 141908.
11. Zhou, W.; Liu, Y. Y.; Yang, Y. Z.; Wu, P. Band Gap Engineering of SnO_2 by Epitaxial Strain: Experimental and Theoretical Investigations. *J. Phys. Chem. C* **2014,** 118, 6448-6453.
12. Heo, S.; Oh, C.; Son, J.; Jang, H. M. Influence of tensile-strain-induced oxygen deficiency on metal-insulator transitions in $NdNiO_{3-\delta}$ epitaxial thin films. *Sci. Rep.* **2017,** 7, 4681.
13. Yokoyama, Y.; Yamasaki, Y.; Taguchi, M.; Hirata, Y.; Takubo, K.; Miyawaki, J.; Harada, Y.; Asakura, D.; Fujioka, J.; Nakamura, M.; Daimon, H.; Kawasaki, M.; Tokura, Y.; Wadati, H. Tensile-Strain-Dependent Spin States in Epitaxial $LaCoO_3$ Thin Films. *Phys. Rev. Lett.* **2018,** 120, 206402.
14. Hirose, T.; Komori, T.; Gushi, T.; Anzai, A.; Toko, K.; Suemasu, T. Strong correlation

between uniaxial magnetic anisotropic constant and in-plane tensile strain in Mn_4N epitaxial films. *AIP Adv.* **2020,** 10, 025117.

15. Cannon, D. D.; Liu, J. F.; Ishikawa, Y.; Wada, K.; Danielson, D. T.; Jongthammanurak, S.; Michel, J.; Kimerling, L. C. Tensile strained epitaxial Ge films on Si(100) substrates with potential application in *L*-band telecommunications. *Appl. Phys. Lett.* **2004,** 84, 906-908.
16. Liu, J. F.; Cannon, D. D.; Wada, K.; Ishikawa, Y.; Danielson, D. T.; Jongthammanurak, S.; Michel, J.; Kimerling, L. C. Deformation potential constants of biaxially tensile stressed Ge epitaxial films on Si(100). *Phys. Rev. B* **2004,** 70, 155309.
17. Liu, J. F.; Cannon, D. D.; Wada, K.; Ishikawa, Y.; Jongthammanurak, S.; Danielson, D. T.; Michel, J.; Kimerling, L. C. Silicidation-induced band gap shrinkage in Ge epitaxial films on Si. *Appl. Phys. Lett.* **2004,** 84, 660-662.
18. Jia, Y.; Wu, B.; Weitering, H. H.; Zhang, Z. Y. Quantum size effects in Pb films from first principles: The role of the substrate. *Phys. Rev. B* **2006,** 74, 035433.
19. Alerhand, O. L.; Vanderbilt, D.; Meade, R. D.; Joannopoulos, J. D. Spontaneous Formation of Stress Domains on Crystal-Surfaces. *Phys. Rev. Lett.* **1988,** 61, 1973-1976.
20. Schulte, F. K. Theory of Thin Metal-Films - Electron-Density, Potentials and Work Function. *Surf. Sci.* **1976,** 55, 427-444.
21. Wang, Z. R.; Zhao, C. X.; Wang, G. Y.; Qin, J.; Xia, B.; Yang, B.; Guan, D. D.; Wang, S. Y.; Zheng, H.; Li, Y. Y.; Liu, C. H.; Jia, J. F. Controllable Modulation to Quantum Well States on β-Sn Islands. *Chin. Phys. Lett.* **2020,** 37, 096801.
